# Diagnostic accuracy of TB-LAMP for pulmonary tuberculosis: a systematic review and meta-analysis

**DOI:** 10.1186/s12879-019-3881-y

**Published:** 2019-03-19

**Authors:** Priya B. Shete, Katherine Farr, Luke Strnad, Christen M. Gray, Adithya Cattamanchi

**Affiliations:** 1 Division of Pulmonary and Critical Care Medicine, University of California San Francisco and Zuckerberg San Francisco General Hospital, 1001 Potrero Avenue, 5K1, San Francisco, CA 94110 USA; 2 Curry International Tuberculosis Center, University of California San Francisco, San Francisco, CA USA; 3 Division of Infectious Diseases, Oregon Health & Science University, 3188 SW Sam Jackson Park Road, Mail Code: L457, Portland, OR 97239 USA; 4 Faculty of Epidemiology and Population Health, London School of Hygiene and Tropical Medicine, Room G30, Keppel Street, London, WC1E 7HT UK

**Keywords:** Tuberculosis, Diagnostic testing, Molecular assay, Point of care

## Abstract

**Background:**

The need for a rapid, molecular test to diagnose tuberculosis (TB) has prompted exploration of TB-LAMP (Eiken; Tokyo, Japan) for use in resource-limited settings. We conducted a systematic review to assess the accuracy of TB-LAMP as a diagnostic test for pulmonary TB.

**Methods:**

We analyzed individual-level data for eligible patients from all studies of TB-LAMP conducted between Jan 2012 and October 2015 to compare the diagnostic accuracy of TB-LAMP with that of smear microscopy and Xpert MTB/RIF® using 3 reference standards of varying stringency. Pooled sensitivity and specificity and pooled differences in sensitivity and specificity were estimated using random effects meta-analysis. Study quality was evaluated using QUADAS-2.

**Results:**

Four thousand seven hundred sixty individuals across 13 studies met eligibility criteria. Methodological quality was judged to be low for all studies. TB-LAMP had higher sensitivity than sputum smear microscopy (pooled sensitivity difference + 13·2, 95% CI 4·5–21·9%) and similar sensitivity to Xpert MTB/RIF (pooled sensitivity difference − 2·5, 95% CI -8·0 to + 2·9) using the most stringent reference standard available. Specificity of TB-LAMP was similar to that of sputum smear microscopy (pooled specificity difference − 1·8, 95% CI -3·8 to + 0·2) and Xpert MTB/RIF (pooled specificity difference 0·5, 95% CI -0·9 to + 1·8).

**Conclusions:**

From the perspective of diagnostic accuracy, TB-LAMP may be considered as an alternative test for sputum smear microscopy. Additional factors such as cost, feasibility, and acceptability in settings that continue to rely on sputum smear microscopy should be considered when deciding to adopt this technology. Xpert MTB/RIF should continue to be preferred in settings where resource and infrastructure requirements are adequate and where HIV co-infection or drug-resistance is of concern.

**Electronic supplementary material:**

The online version of this article (10.1186/s12879-019-3881-y) contains supplementary material, which is available to authorized users.

## Background

Better diagnostics are essential for achieving global tuberculosis (TB) elimination targets. In 2013, Xpert MTB/RIF® (Xpert) (Cepheid, Sunnyvale, CA, USA) became the first molecular TB test endorsed by the World Health Organization (WHO), and there has since been considerable investment in its scale-up [1]. However, relatively high device and consumable costs, infrastructure requirements, and need for continuous instrument maintenance remain obstacles to use of Xpert as a point-of-care test in peripheral health centers where the majority of TB patients initially present for care.

To expand the availability of molecular testing for TB, Eiken Chemical Co., Ltd. developed a commercial version of loop-mediated isothermal amplification (LAMP), a technique in which nucleic acid amplification occurs under isothermal conditions. LAMP is based on auto-cycling, strand displacement DNA synthesis performed by a DNA polymerase with high strand displacement activity and two specially designed inner and two outer primers [2, 3]. Eiken’s Loopamp MTBC Detection Kit (TB-LAMP) targets the *gyrB* and *IS* regions of the *Mycobacterium tuberculosis* (MTB) complex genome. Detection of amplified product is based on turbidity visualized with the naked eye or under ultraviolet (UV) light after 15–60 min [4–7].

To inform WHO guideline development, we conducted a systematic review of studies evaluating the diagnostic accuracy of TB-LAMP using the latest assay kit and protocol. The primary objective was to evaluate the diagnostic accuracy of TB-LAMP if used as an alternative test for sputum smear microscopy among adults suspected of having pulmonary TB. In addition, we sought to determine the diagnostic accuracy of TB-LAMP if used as an alternative test for microscopy among adults with HIV infection or as an add-on test for adults with negative sputum smear microscopy results, and the proportion of indeterminate/invalid TB-LAMP results.

## Methods

We followed standard guidelines and methods for systematic reviews and meta-analyses of diagnostic tests [8]. We developed four PICO style research questions to inform this review. First, what is the diagnostic accuracy of TB-LAMP for detection of pulmonary TB in reference to mycobacterial culture if used as an alternative test for sputum smear microscopy among all adults and among HIV-infected adults? Second, what is the diagnostic accuracy of TB-LAMP for detection of pulmonary TB in reference to mycobacterial culture if used as an add-on test among sputum smear-negative adults? Third, what is the difference in diagnostic accuracy between TB-LAMP and Xpert for detection of pulmonary TB in reference to mycobacterial culture among all adults? And finally, what is the proportion of indeterminate/invalid results when TB-LAMP is used to detect pulmonary TB among all adults and among HIV-infected adults?

### Search strategy

To perform a study of TB-LAMP, investigators must order kits directly from Eiken. Therefore, a list of such studies was requested from Eiken. To confirm the list provided was complete, we performed a search in Google Scholar and PubMed using the terms “TB LAMP”, “TB-LAMP”, and “tuberculosis LAMP”. To meet the deadline for WHO guideline development in January 2016, we only included studies completed by October 1, 2015.

### Study and participant selection

We included all studies which 1) evaluated the Eiken TB-LAMP kit on sputum samples from adult presumptive TB patients; 2) were conducted in an intermediate or high TB burden country as defined by WHO; and 3) were conducted after January 1, 2012 using the final protocol, training, and TB-LAMP kits approved by Eiken. We excluded studies that 1) did not exclude patients on TB treatment within 60 days of enrollment; 2) did not perform speciation testing to confirm presence of *Mycobacterium tuberculosis* (MTB) complex in positive cultures; or 3) performed TB-LAMP on frozen specimens.

Authors of eligible studies provided individual participant data. We excluded individual participants who were 1) less than 18 years of age; 2) did not have results of speciation testing for MTB; 3) had a positive culture but speciation testing identified only non-tuberculous mycobacteria (NTM); 4) had a documented history of prior TB; 5) had TB-LAMP testing performed on non-sputum samples; 6) had TB-LAMP testing done with a total reaction volume of < 25 μL; or 7) could not be classified as TB-positive or TB-negative based on the reference standard definitions described below. When comparing TB-LAMP to Xpert, we also excluded individual participants for whom Xpert was performed on frozen samples or valid results were unavailable for both TB-LAMP and Xpert.

### Quality assessment

We used the Assessment of Diagnostic Accuracy Studies (QUADAS-2) tool to assess the methodological quality of eligible studies [9]. Specific yes/no signaling questions were tailored for each QUADAS-2 domain.

### Index tests

Studies recorded LAMP and Xpert results as negative, positive or indeterminate/invalid in accordance with manufacturer recommendations. We standardized sputum smear microscopy results across studies by considering only direct ZN (Ziehl-Neelsen) and direct FM (fluorescence microscopy) results, considering only the first two smear results if more than two direct ZN or direct FM results were available, and defining patients to be sputum smear-positive if ≥1 acid-fast bacillus (AFB) was identified in any sputum smear.

### Reference standard

We used three hierarchical mycobacterial culture-based reference standards to account for differences in the number of cultures performed and results available. Patients were classified as having TB if one or more cultures were positive and confirmed to be MTB complex by speciation testing. Patients were classified as not having TB if there were no positive cultures and at least 1) two negative cultures performed on different sputum samples (Standard 1); 2) two negative cultures performed on one sputum sample (Standard 2); or 3) one negative culture (Standard 3). The three reference standards allowed for a trade-off between yield of TB diagnosis (highest with Standard 1 and lowest with Standard 3) and number of studies/participants included in each analysis (lowest with Standard 1 and highest with Standard 3).

### Statistical analysis

For all review questions, we assessed heterogeneity visually with forest plots and statistically with the χ^2^ test for heterogeneity and the *I*^2^ test of inconsistency [10, 11]. When four or more studies were available, we generated pooled summary estimates of sensitivity/specificity using hierarchical summary receiver operating characteristic (HSROC) analysis. We plotted these estimates along with 95% confidence regions in ROC space. Pooled differences in sensitivity/specificity between TB-LAMP and Xpert and pooled proportion of indeterminate/invalid TB-LAMP results were generated using random effects meta-analysis. For the primary review question of TB-LAMP accuracy if used as an alernative test for smear microscopy, we explored potential reasons for heterogeneity by performing sub-group analyses based on the health system level (reference lab, microscopy center, or hospital-affiliated clinics) at which the study was conducted and study quality (high-quality studies across all domains and within each domain of QUADAS-2).

## Results

**Fig. 1:**
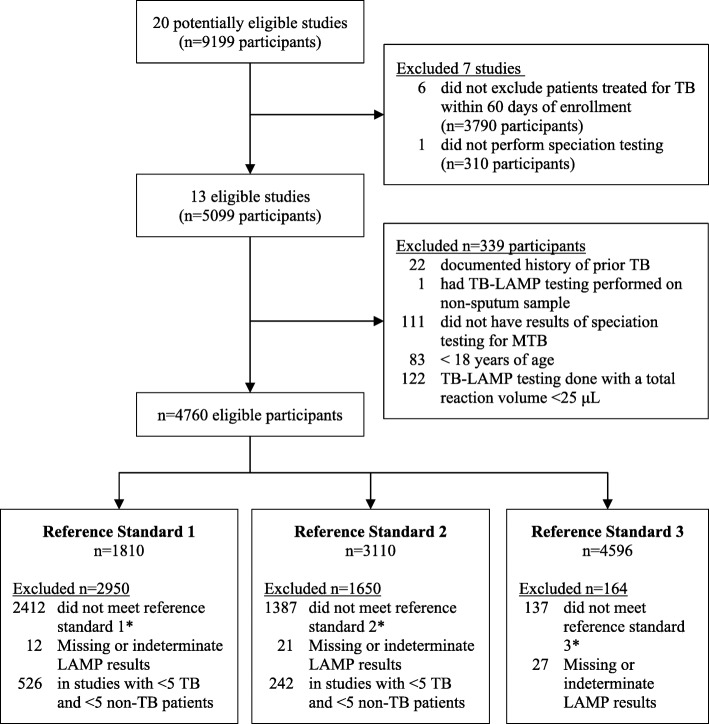
Study and participant selection flow diagram. Of 20 potentially eligible studies, 13 met study-level eligibility criteria. The 13 eligible studies included 5099 participants, of whom 339 (7%) did not meet participant-level eligibility criteria. Of the 4760 eligible participants, 1810 (38%) were included in the analysis for reference standard 1, 3110 (65%) for reference standard 2, and 4596 (97%) for reference standard 3. Abbreviations: TB, Tuberculosis; MTB, *Mycobacterium tuberculosis*; μL, microliter

Of 20 studies identified by Eiken at the time of this review, 13 met criteria for inclusion (Fig. 1): four evaluation (EVAL) studies were conducted by the Foundation for Innovative New Diagnostics (FIND) in reference labs; one demonstration (DEMO) study was conducted by FIND in a peripheral microscopy center; seven studies were commissioned by FIND through a request for applications (RFA); and one study was sponsored by Eiken [12–14]. Authors of included studies submitted individual data for 5099 participants, of whom 4760 were eligible for analysis (Additional file 1: Table S1).

Of the included studies, four were conducted at reference laboratories, six at hospital and/or university-affiliated clinics, and three at peripheral microscopy centers (Table 1). Study participants were majority male with a median age of 40 (IQR 29–54) years. More than 10% of participants were known to be HIV-positive in four of 13 studies (S. Africa EVAL, Malawi RFA, Uganda RFA, Ivory Coast RFA). The proportion of patients with culture-positive TB was 20–40% in most studies, but was notably lower (8–15%) in three studies (India DEMO, India RFA, Vietnam RFA) and higher (66%) in one study (Vietnam EVAL). The proportion of patients with smear-negative TB ranged widely from 13 to 59%.

**Table 1:** Study characteristics

Study	Health system level	Microscopy type	TB culture type	MGIT contamination rate	Tests done on stored sputum	Xpert specimen type	Median Age (IQR)	Female	HIV^a^	Culture-positive TB^b^	Smear- negative TB^c^
Evaluation Studies											
Brazil	Reference Lab	Direct ZN ×2	2x MGIT, 2x LJ	0%	Xpert Culture	Frozen Processed	48 (35–60)	40%	0.4%	32%	25%
Peru	Reference Lab	Direct ZN ×2	2x MGIT, 2x LJ	1·3%	Xpert Culture	Fresh Processed	43 (28–56)	50%	1.0%	22%	42%
South Africa	Reference Lab	Direct ZN ×2	2x MGIT, 2x LJ	3·1%	Xpert Culture	Fresh Processed	39 (29–47)	34%	35%	26%	51%
Vietnam	Reference Lab	Direct ZN ×2	2x MGIT, 2x LJ	0%	Xpert Culture	Frozen Processed	39 (26–50)	30%	2%	66%	42%
Demonstration Study											
India	Microscopy center	Direct ZN ×2	MGIT, LJ	6·5%	Culture	None	40 (27–51)	35%	2%	11%	25%
Request for Application (RFA) Studies											
India	University-affiliated DOTS clinic	Direct ZN × 1	MGIT	11·5%	Xpert Culture	Frozen Processed	43 (29–55)	37%	2%	15%	54%^3^
Vietnam	Microscopy center	Direct ZN × 2	MGIT	0%	Xpert Culture	Fresh Processed	60 (52–70)	42%	0.5%	8%	59%
Malawi	Microscopy center	Direct FM × 1	MGIT, LJ	8·7%	Xpert Culture	Fresh Direct	35 (26–41)	48%	44%	16%	15%^3^
Tanzania	District Hospital TB clinic	Direct FM × 2	MGIT, LJ	5·1%	Culture	Fresh Direct	37 (28–46)	45%	6%	29%	28%
Uganda	District Hospital outpatient clinic	Direct FM ×2	2x MGIT, 2x LJ	1·3%	Culture	Fresh Direct	43 (30–54)	43%	48%	31%	45%
Ivory Coast	District Hospital outpatient clinic	Direct ZN ×2	MGIT	6·0%	Xpert Culture	Fresh Processed	38 (28–44)	51%	12%	33%	13%
Madagascar	University-affiliated DOTS clinic	Direct FM ×2	2x LJ	---	Xpert Culture	Fresh Direct	42 (29–52)	40%	---	37%	27%
Sponsored Study											
Haiti (Kaku, et al)	Urban Hospital outpatient clinic	Direct FM ×2	3x MGIT	0%	Culture	None	---	---	---	34%	23%

--- information not available

Abbreviations: *TB* tuberculosis, *MGIT* Mycobacterial Growth Indicator Tube, *ZN* Ziehl-Neelsen, *FM* fluorescence microscopy, *LJ* Lowenstein-Jensen

^a^HIV unknown counted as negative. Reflects proportion of study population known to be HIV positive

^b^Reference standard 3 used for all studies in this calculation

^c^Smear microscopy results based on analysis of 1 smear only

### Methodological quality

**Fig. 2:**
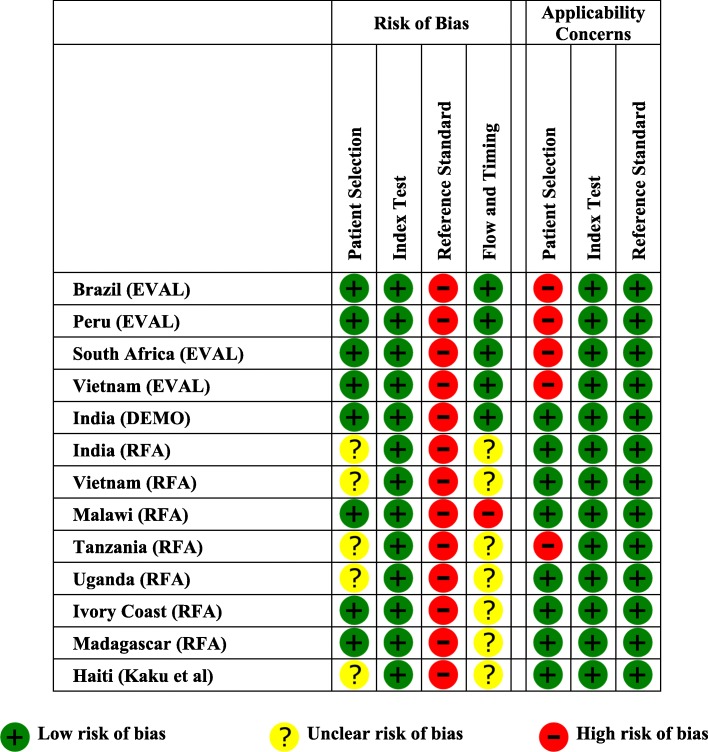
Study quality assessment using QUADAS-2. Based on QUADAS-2 assessment, the risk of bias was judged to be unclear in 5 (38%) studies due to patient selection issues, high for all studies due to an inadequate reference standard, and either high in 1 (8%) study or unclear in 7 studies (54%) due to flow and timing issues. In addition, applicability concerns were high in 5 (38%) studies due to patient selection issues

We considered overall risk of bias to be high due to problems with the culture-based reference standard (all 13 studies), unclear patient selection (five studies), and flow and timing concerns (eight studies) (Fig. 2). We found applicability concerns to be low for index tests and the reference standard; however, we judged five studies to have high applicability concerns for patient selection because they were conducted at referral laboratories/centers or because enrollment involved screening of patients by a pulmonary specialist (Additional file 1: Table S2).

### TB-LAMP accuracy if used as an alternative test for smear microscopy

**Fig. 3:**
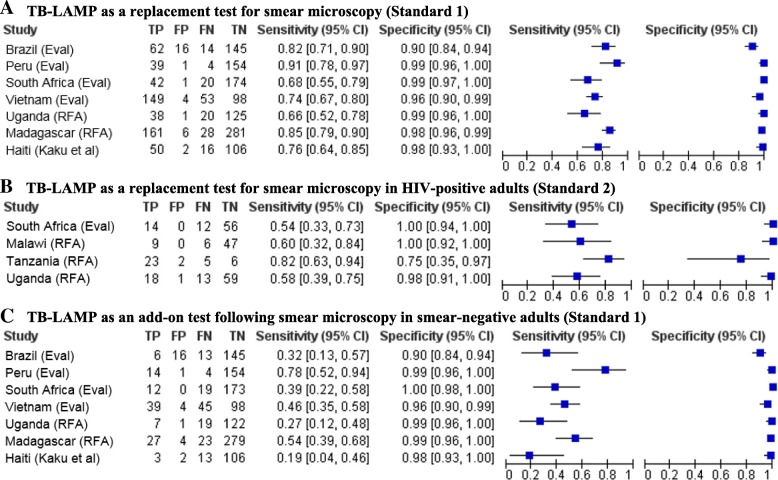
Forest plots of TB-LAMP diagnostic accuracy, best reference standard. The figures show the sensitivity and specificity of TB-LAMP in individual studies in reference to the best available reference standard for TB-LAMP as an alternative test for smear microscopy in all patients (Panel 3A), TB-LAMP as an alternative test for smear microscopy in HIV-positive adults (Panel 3B), and TB-LAMP as an add-on test following smear microscopy (Panel 3C). All reference standards classify patients as having TB if ≥1 positive culture was confirmed as *M. tuberculosis* by speciation testing. To be classified as not having TB, patients were required to have no positive and at least 1) two negative cultures on two different sputum specimens (Standard 1); or 2) two negative cultures on the same or different sputum specimens (Standard 2). Visual inspection of all three forest plots indicates considerable heterogeneity in sensitivity estimates but less heterogeneity in specificity estimates

Sensitivity of TB-LAMP in individual studies ranged from 66 to 91% with Standard 1 (Fig. 3), 62–91% with Standard 2 and 48–100% with Standard 3 (Additional file 1: Figure S1). We found significant heterogeneity in sensitivity estimates, both from visual inspection of forest plots and statistical testing (*I*^2^ 72–94%, *p* < 0·003 for all reference standards). Pooled sensitivity of TB-LAMP ranged from 77·7% (95% CI 71·2–83·0) when using Standard 1 to 80·3% (95% CI 70·3–87·5) when using Standard 3 (Table 2 and Additional file 1: Figure S2). TB-LAMP was more sensitive than sputum smear microcopy, with the sensitivity difference ranging from 7·1% (95% CI 1·4–12·9) when using Standard 1 to 13·2% (95% CI 4·5–21·9) when using Standard 3.

**Table 2:** Pooled sensitivity and specificity of TB-LAMP

Reference Standard	Pooled Sensitivity	Pooled Specificity
TB-LAMP accuracy if used as an alternative test for smear microscopy in all adults		
Standard 1^a^	77·7 (71·2–83·0)	98·1 (95·7–99·2)
Standard 2^a^	76·0 (69·9–81·2)	98·0 (96·0–99·0)
Standard 3^a^	80·3 (70·3–87·5)	97·7 (96·1–98·7)
TB-LAMP accuracy if used as an alternative test for smear microscopy in HIV-positive adults		
Standard 1^a^	N/A (< 4 studies)	N/A (< 4 studies)
Standard 2^a^	63·8 (49·0–76.4)	98·8 (85·1–99·9)
Standard 3^a^	73·4 (51·9–87·6)	95·0 (64·0–99·5)
TB-LAMP accuracy if used as an add-on test in smear-negative adults		
Standard 1^a^	42·1 (30·0–55·3)	98·4 (95·9–99·4)
Standard 2^a^	42·2 (27·9–57·9)	98·0 (96·0–99·0)
Standard 3^a^	40·3 (27·9–54·0)	97·7 (96·1–98·6)
TB-LAMP accuracy in studies comparing to Xpert^b^		
Standard 1^a^	78·0 (66·6–86·4)	98·9 (97·4–99·6)
Standard 2^a^	74·1 (64·1–82·2)	98·8 (96·8–99·6)
Standard 3^a^	75·8 (63·2–85·0)	98·2 (96·0–99·2)
Xpert accuracy in studies comparing to TB-LAMP^b^		
Standard 1^a^	81·1 (70·6–88·5)	98·2 (95·9–99·2)
Standard 2^a^	80·4 (73·4–85·9)	97·4 (94·9–98·7)
Standard 3^a^	84·0 (75·6–90·0)	97·2 (94·4–98·6)

^a^All reference standards classify patients as having TB if ≥1 positive culture was confirmed as *M. tuberculosis* by speciation testing. To be classified as not having TB, patients were required to have no positive and at least 1) two negative cultures on two different sputum specimens (Standard 1); 2) two negative cultures on the same or different sputum specimens (Standard 2); or 3) at least one negative culture (Standard 3)

^b^Data restricted to study participants who had valid results for both TB-LAMP and Xpert and testing performed on non-frozen specimens

Abbreviations: *N/A* not applicable

Specificity of TB-LAMP in individual studies ranged from 90 to 99% with Standard 1 (Fig. 3), and 90–100% with Standards 2 and 3 (Additional file 1: Figure S1). Visual inspection of forest plots indicated heterogeneity in specificity estimates was less than for sensitivity estimates, but was still significant (*I*^2^ 61–78%, *p* < 0·03 for all reference standards). Pooled specificity of TB-LAMP ranged from 97·7% (95% CI 96·1–98·7) when using Standard 3 to 98·1% (95% CI 95·7–99·2) when using Standard 1 (Table 2 and Additional file 1: Figure S2). TB-LAMP had similar specificity to sputum smear microscopy, with specificity difference ranging from − 1·8% (95% CI -3·8–0·2) when using Standard 1 to − 1·3% (95% CI -3·1% to + 0·4) when using Standard 3.

Sub-group analyses did not reduce heterogeneity in pooled sensitivity and specificity estimates. Pooled sensitivity was 78·0% (95% CI 68·9–85·0) among studies conducted in reference labs and 86·8% (95% CI 67·9–95·4) among studies conducted in hospital-affiliated clinics, and there was significant heterogeneity within both sub-groups (*I*^2^ 74—96%, *p* < 0·01 for all reference standards) (Additional file 1: Table S3). Among studies rated as high-quality for patient selection, pooled sensitivity was 84·2% (95% CI 71·1–92·0) and pooled specificity 98·1% (95% CI 94·3–99·4), but there was significant heterogeneity across studies (*I*^2^ 83%, p < 0·001 for sensitivity and *I*^2^ 87%, p < 0·001 for specificity). Pooled estimates could not be obtained for studies conducted at microscopy centers and studies rated as high-quality for reference standard because there were less than four studies in these sub-groups.

### TB-LAMP accuracy if used as an alternative test for smear microscopy in HIV-infected adults

Pooled sensitivity of TB-LAMP was lower among HIV-infected adults than all adults, ranging from 63·8% (95% CI 49·0–76·4) with Standard 2 to 73·4 (95% CI 51·9–87·6) with Standard 3 (Table 2 and Additional file 1: Figure S3). Pooled specificity was low with Standard 3 (95·0, 95% CI 64·0–99·5) but high with Standard 2 (98·8, 95% CI 85·1–99·9). There was considerable heterogeneity in sensitivity (*I*^2^ 86%, *p* < 0·001) and specificity (*I*^2^ 86%, p < 0·001) estimates with Standard 3, but not Standard 2 (*I*^2^ 54%, *p* = 0·09 for sensitivity and *I*^2^ 0%, p = 0·42 for specificity) (Fig. 3). There were insufficient studies (*N* = 2) to perform meta-analysis using Standard 1.

### TB-LAMP accuracy if used as an add-on test in smear-negative adults

As expected, pooled sensitivity of TB-LAMP was lower among smear-negative adults than among all adults, ranging from 40·3% (95% CI 27·9–54·0) with Standard 3 to 42·2% (95% CI 27·9–57·9) with Standard 2 (Table 2 and Additional file 1: Figure S4). Pooled specificity of TB-LAMP among smear-negative adults was similar to that observed among all adults, ranging from 97·7% (95% CI 96·1–98·6) with Standard 3 to 98·4% (95% CI 95·9–99·4) with Standard 1. There was greater heterogeneity in sensitivity estimates than in specificity estimates across studies (Fig. 3).

### Comparison of diagnostic accuracy of TB-LAMP and Xpert

Xpert sensitivity across individual studies ranged from 65 to 97% between the three reference standards and specificity ranged from 90 to 100% (Additional file 1: Figure S5). TB-LAMP sensitivity ranged from 48 to 93% between reference standards, and specificity ranged from 94 to 100% (Additional file 1: Figure S6). The difference in sensitivity between TB-LAMP and Xpert in individual studies ranged from − 14 to + 3% for Standard 1, − 15 to + 3% for Standard 2, and − 36 to + 3% for Standard 3 (Additional file 1: Figure S7). TB-LAMP had similar sensitivity compared to Xpert using Standard 1 (pooled sensitivity difference − 2·5% (95% CI -8·0 to + 2·9) but lower sensitivity when using Standard 3 (− 6·9, 95% CI -12·8 to − 1·0) (Table 3). Difference in specificity between TB-LAMP and Xpert in individual studies ranged from − 1 to + 3% for Standard 1, − 1 to + 4% for Standard 2, and − 3 to + 5% for Standard 3 (Additional file 1: Figure S8). There was no difference in specificity of TB-LAMP and Xpert regardless of reference standard used (Table 3). Heterogeneity varied depending on the reference standard used, with minimal heterogeneity in sensitivity and specificity differences across studies with Standard 1, and significant heterogeneity with Standard 3 (Additional file 1: Figure S7 and Figure S8).

**Table 3:** TB-LAMP versus Xpert: Pooled Sensitivity and Specificity differences

Reference standard	Pooled sensitivity difference^b^	Pooled specificity difference^b^
Standard 1^a^	-2·5 (− 8·0 to + 2·9)	0·5 (− 0·9 to + 1·8)
Standard 2^a^	− 6·0 (− 12·1 to + 0·1)	1·0 (− 0·3 to + 2·4)
Standard 3^a^	− 6·9 (− 12·8 to − 1·0)	1·1 (− 0·7 to + 2·8)

^a^All reference standards classify patients as having TB if ≥1 positive culture was confirmed as *M. tuberculosis* by speciation testing. To be classified as not having TB, patients were required to have no positive and at least 1) two negative cultures on two different sputum specimens (Standard 1); 2) two negative cultures on the same or different sputum specimens (Standard 2); or 3) at least one negative culture (Standard 3)

^b^Positive difference favors TB-LAMP

### Indeterminate/invalid TB-LAMP results

The proportion of indeterminate TB-LAMP results was 0% in 11 studies and 1% in two studies (Additional file 1: Figure S9). There was minimal heterogeneity across studies (*I*^2^ 28%, *p* = 0·25). Pooled proportion of indeterminate TB-LAMP results was < 0.1% (95% CI 0–0). Results were similar among HIV-infected adults; pooled proportion of indeterminate TB-LAMP results in this sub-group was < 0.1% (95% CI 0–1).

## Discussion

This systematic review identified 13 studies conducted in intermediate to high TB burden countries evaluating the accuracy of TB-LAMP performed directly on sputum samples for diagnosis of pulmonary TB. TB-LAMP had moderate sensitivity (pooled sensitivity 77·7, 95% CI 71·2–83·0) and high specificity (98·1, 95% CI 95·7–99·2) when using the most stringent culture-based reference standard. Sensitivity was lower among HIV-infected adults (pooled sensitivity 63·8, 95% CI 49·0–76.4), likely due to a higher proportion of smear-negative TB in this population. Among all adults, TB-LAMP would identify slightly less than half of all smear-negative TB patients (pooled sensitivity 42·1, 95% CI 30·0–55·3) if used as an add-on test following sputum smear microscopy. Diagnostic accuracy was comparable to that of Xpert, with no significant differences in pooled sensitivity (− 2·5, 95% CI -8·0 to + 2·9) or pooled specificity (0·5, 95% CI -0·9 to + 1·8) using the most stringent reference standard. Finally, this review found indeterminate TB-LAMP results were extremely uncommon (pooled proportion < 0.1, 95% CI 0–0) (Additional file 1: Figure S9). Overall, these data support a potential role for TB-LAMP in the diagnosis of pulmonary TB in intermediate- to high-burden countries where smear-microscopy is still the predominant mode of TB diagnosis.

The target product profile for an alternative test for smear microscopy recommends a sensitivity of at least 80% and specificity of at least 98% [15]. TB-LAMP very nearly meets these criteria, although sensitivity was below the recommended minimum of 60% for smear-negative TB and 99% for smear-positive TB. Nonetheless, TB-LAMP was consistently more sensitive than sputum smear microscopy in individual studies, and pooled sensitivity difference was 7·1%–13·2% in favor of TB-LAMP depending on reference standard used. Thus, it can be expected that use of TB-LAMP as an alternative for sputum smear microscopy would lead to more TB cases being identified while keeping false-positive results to an acceptable minimum. As discussed further below, a better reference standard would have likely further increased difference in sensitivity and minimized any difference in specificity between TB-LAMP and smear microscopy.

Although more accurate than microscopy, pooled sensitivity of TB-LAMP is lower than has been reported for Xpert (89, 95% CI 85–92] [16]. However, in head-to-head comparisons, the sensitivity difference between TB-LAMP and Xpert was not statistically significant except when using the least stringent reference standard. Although more data is needed to confirm whether TB-LAMP is as sensitive as Xpert, it is important to consider TB-LAMP has a different end-user profile. It is less costly to deploy and has fewer infrastructure requirements (e.g., stable power), but has higher training requirements due to less automation. Even if confirmed to be less sensitive than Xpert, TB-LAMP may have a role at health centers that have personnel with adequate technical skills but insufficient resources or infrastructure to deploy Xpert. In addition, the specificity of TB-LAMP was as high or higher than that of Xpert, further supporting its use as an alternative test for microscopy in settings without access to Xpert.

There are several limitations to the evidence identified in this review. Most significantly, an inadequate reference standard was used across all studies, likely leading to classification of patients with TB as not having TB. With a better reference standard, it can be expected that some false-positive TB-LAMP results would be re-classified as true positives, leading to improved sensitivity and specificity. Some true negative TB-LAMP results could also be re-classified as false negatives, leading to lower sensitivity and specificity. Our findings suggest the former is more likely, given that TB-LAMP specificity improved with a more stringent reference standard (i.e., when moving from Standard 3 to Standard 1). A better reference standard may also have increased the sensitivity difference while further minimizing the specificity difference observed between Xpert and TB-LAMP due to the higher number of false-positive Xpert results. Another key limitation is that the included studies may not accurately reflect the introduction of TB-LAMP under programmatic conditions. Only 3 studies were conducted at peripheral health centers and Eiken provided extensive training to sites included in all studies. Further operational research is needed to characterize TB-LAMP performance under typical implementation conditions as an alternative test for microscopy at peripheral health centers. Finally, the data used in this analysis was taken from studies sponsored by either a non-governmental organizations (NGOs) with interest in the product or the manufacturer (Eiken), raising potential concerns about bias due to conflict of interest. To overcome this limitation, this systematic review and meta-analysis were the result of independent analysis of individual level data taken from these studies. PICO questions and reference standards were devised by the authors in conjunction with the World Health Organization as part of their guideline development process.

In summary, this systematic review supports use of TB-LAMP as a potential alternative test for smear microscopy for diagnosis of pulmonary TB in intermediate- to high-burden countries, particularly in settings where Xpert testing is not feasible. The results of this review have influenced development of WHO guidelines related to the use of TB-LAMP, which now conditionally recommend the use of TB-LAMP as an alternative test for smear microscopy or as an add-on test for smear negative patients, but do not recommend its use in settings where molecular testing such as Xpert is readily available [17]. However, additional studies following standardized protocols and including a high-quality reference standard (liquid culture results on at least two samples) are needed to better inform National TB Programmes of the relative performance of TB-LAMP versus Xpert in programmatic settings. Cost effectiveness analysis conducted as part of WHO guideline development on the use of TB-LAMP demonstrated lower per test cost, operational costs, budgetary costs and favorable incremental cost effectiveness ratios compared to Xpert in a few countries [18]. As with feasibility assessments, additional cost and cost effectiveness analyses in programmatic settings are required to further inform context-specific uptake of TB-LAMP. The evidence to date, along with increased automation and the ability to identify rifampin resistance, suggests Xpert should remain the preferred diagnostic when sufficient financial resources and infrastructure can support its use.

## Conclusions

Although the performance of TB-LAMP has been evaluated in several studies worldwide with variable results, we report the first standardized evaluation of the results of these individual studies to inform policy guidance. The findings of our systematic review and meta-analysis show that TB-LAMP has the potential to be a useful diagnostic test for pulmonary TB, but that additional high quality studies in programmatic conditions should be done to strengthen the case for its use. TB-LAMP performed better than sputum smear microscopy (more sensitive and as specific) in the diagnosis of pulmonary tuberculosis and performed similar to Xpert (similar sensitivity and specificity). The results of this study provided the basis for the WHO’s guidelines on the use of TB-LAMP for the diagnosis of pulmonary TB, which recommend that TB-LAMP can be used as an alternative for microscopy for the diagnosis of pulmonary TB in adults, and can be considered as an add-on test to microscopy particularly in sputum smear-negative adults with TB symptoms in settings where Xpert testing is not available and where drug-resistance or HIV co-infection are not of concern [17].

## Additional file

Additional file 1: **Figure S1.** Forrest plots of TB-LAMP diagnostic accuracy, additional reference standards. The figures show the sensitivity and specificity of TB-LAMP in individual studies in reference to all additional reference standards not judged best available for TB-LAMP as an alternative test for smear microscopy in all patients (Panel S1A and Panel S1B), TB-LAMP as an alternative test for smear microscopy in HIV-positive adults (Panel S1C), and TB-LAMP as an add-on test following smear microscopy (Panel S1D and Panel S1E). All reference standards classify patients as having TB if ≥1 positive culture was confirmed as *M. tuberculosis* by speciation testing. To be classified as not having TB, patients were required to have no positive and at least 1) two negative cultures on two different sputum specimens (Standard 1); 2) two negative cultures on the same or different sputum specimens (Standard 2); or 3) at least one negative culture (Standard 3). Visual inspection of all three forest plots indicates considerable heterogeneity in sensitivity estimates but less heterogeneity in specificity estimates. **Figure S2.** TB-LAMP as an alternative for sputum smear microscopy: Summary Receiver Operating Characteristic (SROC) curves. The figure shows SROC curves for TB-LAMP (green line), individual study estimates (grey circle), pooled estimates (red square), and the 95% confidence region for pooled estimates (yellow dotted line) when using 3 culture-based reference standards. All reference standards classify patients as having TB if ≥1positive culture was confirmed as *M. tuberculosis* by speciation testing. To be classified as not having TB, patients were required to have no positive and at least 1) two negative cultures on two different sputum specimens (Standard 1); 2) two negative cultures on the same or different sputum specimens (Standard 2); or 3) at least one negative culture (Standard 3). **Figure S3.** TB-LAMP as an alternative test for smear microscopy in HIV-positives: Summary Receiver Operating Characteristic (SROC) curves. The figure shows SROC curves for TB-LAMP (green line), individual study estimates (grey circle), pooled estimates (red square), and the 95% confidence region for pooled estimates (yellow dotted line) when using 2 culture-based reference standards (no studies qualified for Standard 1). All reference standards classify patients as having TB if ≥1positive culture was confirmed as *M. tuberculosis* by speciation testing. To be classified as not having TB, patients were required to have no positive and at least 1) two negative cultures on the same or different sputum specimens (Standard 2); or 2) at least one negative culture (Standard 3). **Figure S4.** TB-LAMP as an add-on test following smear microscopy: Summary Receiver Operating Characteristic (SROC) curves. The figure shows SROC curves for TB-LAMP (green line), individual study estimates (grey circle), pooled estimates (red square), and the 95% confidence region for pooled estimates (yellow dotted line) when using 3 culture-based reference standards. All reference standards classify patients as having TB if ≥1positive culture was confirmed as *M. tuberculosis* by speciation testing. To be classified as not having TB, patients were required to have no positive and at least 1) two negative cultures on two different sputum specimens (Standard 1); 2) two negative cultures on the same or different sputum specimens (Standard 2); or 3) at least one negative culture (Standard 3). **Figure S5.** TB-LAMP vs. Xpert MTB/RIF: Forest plots of Xpert MTB/RIF diagnostic accuracy. The figure shows forest plots of Xpert MTB/RIF sensitivity and specificity in reference to 3 culture-based reference standards for individual studies. All reference standards classify patients as having TB if ≥1positive culture was confirmed as *M. tuberculosis* by speciation testing. To be classified as not having TB, patients were required to have no positive and at least 1) two negative cultures on two different sputum specimens (Standard 1); 2) two negative cultures on the same or different sputum specimens (Standard 2); or 3) at least one negative culture (Standard 3). **Figure S6.** TB-LAMP vs. Xpert MTB/RIF: Forest plots of TB-LAMP diagnostic accuracy. The figure shows forest plots of TB-LAMP sensitivity and specificity in reference to 3 culture-based reference standards for individual studies. All reference standards classify patients as having TB if ≥1positive culture was confirmed as *M. tuberculosis* by speciation testing. To be classified as not having TB, patients were required to have no positive and at least 1) two negative cultures on two different sputum specimens (Standard 1); 2) two negative cultures on the same or different sputum specimens (Standard 2); or 3) at least one negative culture (Standard 3). **Figure S7.** TB-LAMP vs. Xpert MTB/Rif: Forest plots of sensitivity difference. The figure shows forest plots of the sensitivity difference between TB-LAMP and Xpert MTB/Rif® for individual studies. The sensitivity of both tests was calculated in reference to 3 culture-based reference standards. All reference standards classify patients as having TB if ≥1positive culture was confirmed as *M. tuberculosis* by speciation testing. To be classified as not having TB, patients were required to have no positive and at least 1) two negative cultures on two different sputum specimens (Standard 1); 2) two negative cultures on the same or different sputum specimens; or 3) at least one negative culture (Standard 3). Visual inspection of forest plots and statistical testing indicate minimal heterogeneity with Standard 1 (*I*^2^ 0%, *p* = 0.41), some heterogeneity with Standard 2 (*I*^2^ 34%, *p* = 0.18), and significant heterogeneity with Standard 3 (*I*^2^ 55%, *p* = 0.03). **Figure S8.** TB-LAMP vs. Xpert MTB/Rif®: Forest plots of specificity difference. The figure shows forest plots of the specificity difference between TB-LAMP and Xpert MTB/Rif® for individual studies. The specificity of both tests was calculated in reference to 3 culture-based reference standards. All reference standards classify patients as having TB if ≥1positive culture was confirmed as *M. tuberculosis* by speciation testing. To be classified as not having TB, patients were required to have no positive and at least 1) two negative cultures on two different sputum specimens (Standard 1); 2) two negative cultures on the same or different sputum specimens (Standard 2); or 3) at least one negative culture (Standard 3). Visual inspection of forest plots and statistical testing indicate minimal heterogeneity with Standard 1 (*I*^2^ 28%, *p* = 0.25) and Standard 2 (*I*^2^ 37%, *p* = 0.16), but significant heterogeneity with Standard 3 (*I*^2^ 72%, *p* = 0.001). **Figure S9.** Proportion of indeterminate TB-LAMP results. The figure shows a forest plot of the proportion of indeterminate TB-LAMP results among all adults for individual studies. Visual inspection of forest plots and statistical testing indicate minimal heterogeneity (*I*^2^ 28%, *p* = 0.25). **Table S1.** Patients included for analysis. **Table S2.** Signaling questions for QUADAS-2 domains. **Table S3.** TB-LAMP as an alternative test for smear microscopy: Exploration of heterogeneity. (DOCX 770 kb)

## Abbreviations

AFB: Acid fast bacilli
CI: Confidence interval
DEMO: Demonstration study
DNA: Deoxyribonucleic acid
EVAL: Evaluation study
FIND: Foundation for Innovative Diagnostics
FM: Fluorescence microscopy
HIV: Human immunodeficiency virus
HSROC: Hierarchical summary reciever operating characteristic
LAMP: Loop-mediated isothermal amplification
MTB: *Mycobacterium tuberculosis*
NTM: Non-tuberculous mycobacteria
QUADAS: Quality Assessment of Studies of Diagnostic Accuracy for Systematic Reviews
RFA: Request for Applications
RIF: Rifampin
TB: Tuberculosis
UV: Ultraviolet
WHO: World Health Organization
ZN: Ziehl-Neelsen
