# Supplementary material for: Diagnostic accuracy of TB-LAMP for pulmonary tuberculosis: a systematic review and meta-analysis
Source: BMC Infect Dis. 2019 Mar 19;19:268. doi: 10.1186/s12879-019-3881-y (PMC6425614; doi:10.1186/s12879-019-3881-y)
Supplement: Supplementary file 1 — Figure S1. Forrest plots of TB-LAMP diagnostic accuracy, additional reference standards. The figures show the sensitivity and specificity of TB-LAMP in individual studies in reference to all additional reference standards not judged best available for TB-LAMP as an alternative test for smear microscopy in all patients (Panel S1A and Panel S1B), TB-LAMP as an alternative test for smear microscopy in HIV-positive adults (Panel S1C), and TB-LAMP as an add-on test following smear microscopy (Panel S1D and Panel S1E). All reference standards classify patients as having TB if ≥1 positive culture was confirmed as M. tuberculosis by speciation testing. To be classified as not having TB, patients were required to have no positive and at least 1) two negative cultures on two different sputum specimens (Standard 1); 2) two negative cultures on the same or different sputum specimens (Standard 2); or 3) at least one negative culture (Standard 3). Visual inspection of all three forest plots indicates considerable heterogeneity in sensitivity estimates but less heterogeneity in specificity estimates. Figure S2. TB-LAMP as an alternative for sputum smear microscopy: Summary Receiver Operating Characteristic (SROC) curves. The figure shows SROC curves for TB-LAMP (green line), individual study estimates (grey circle), pooled estimates (red square), and the 95% confidence region for pooled estimates (yellow dotted line) when using 3 culture-based reference standards. All reference standards classify patients as having TB if ≥1positive culture was confirmed as M. tuberculosis by speciation testing. To be classified as not having TB, patients were required to have no positive and at least 1) two negative cultures on two different sputum specimens (Standard 1); 2) two negative cultures on the same or different sputum specimens (Standard 2); or 3) at least one negative culture (Standard 3). Figure S3. TB-LAMP as an alternative test for smear microscopy in HIV-positives: Summary Receiver [file 12879_2019_3881_MOESM1_ESM.docx]

**SUPPLEMENTARY MATERIALS**

**Supplementary Figure 1. Forrest plots of TB-LAMP diagnostic accuracy, additional reference standards.**

**S1A. TB-LAMP as a replacement test for smear microscopy (Standard 2)**

**
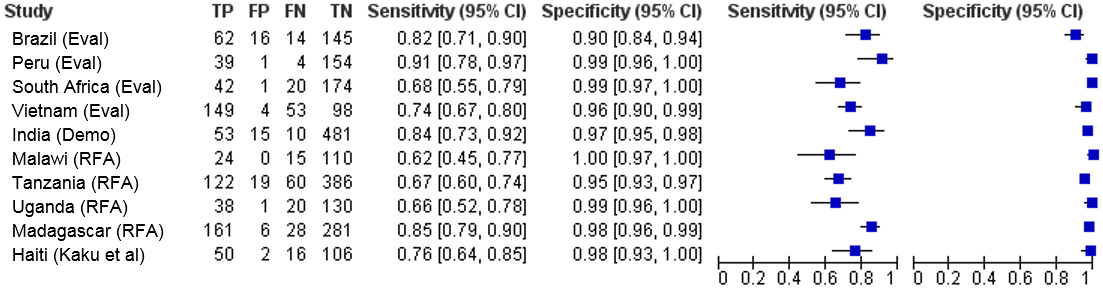
**

**S1B. TB-LAMP as a replacement test for smear microscopy (Standard 3)**

**
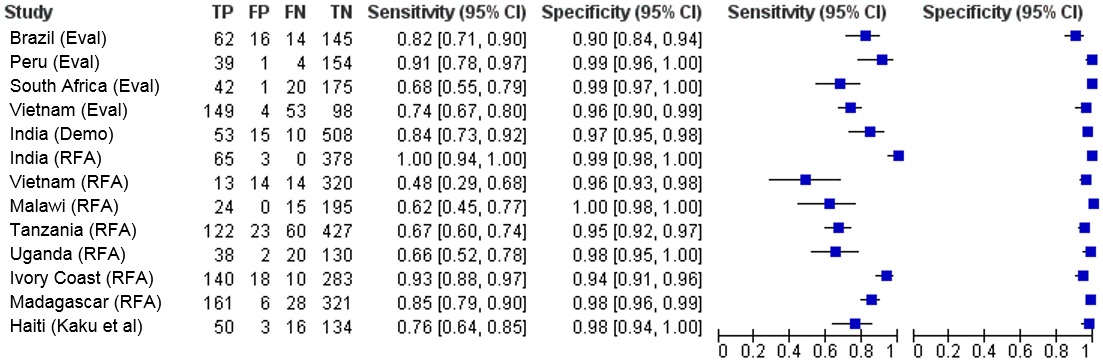
**

**S1C. TB-LAMP as a replacement test for smear microscopy in HIV-positive adults (Standard 3)**

**
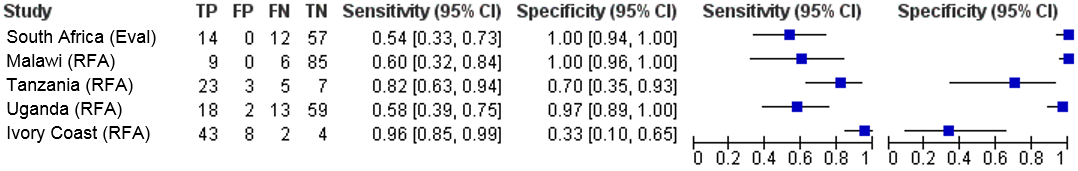
**

**S1D. TB-LAMP as an add-on test following smear microscopy (Standard 2)**

**
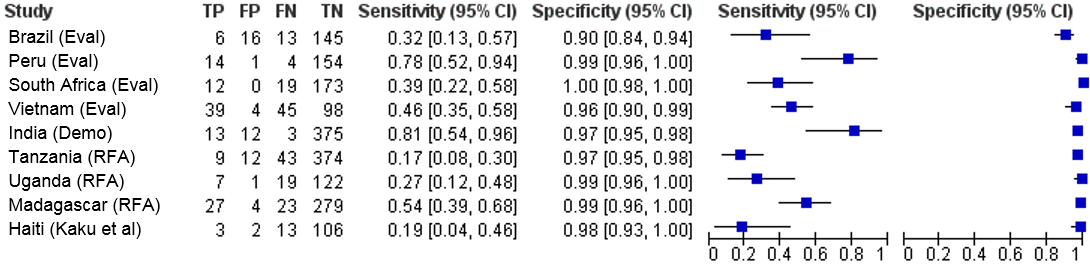
**

**S1E. TB-LAMP as an add-on test following smear microscopy (Standard 3)**

**
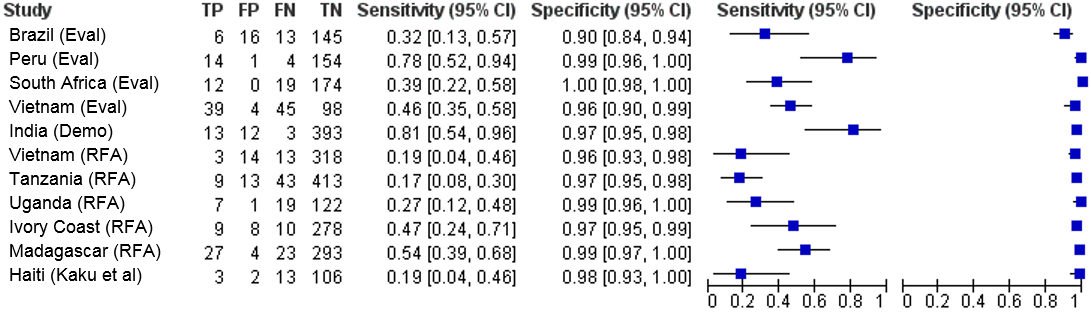
**

**Supplementary Figure 2. TB-LAMP as a replacement for sputum smear microscopy: Summary Receiver Operating Characteristic (SROC) curves.**

| \| **S2A: Standard 1**   \| **S2B: Standard 2**   \| **S2C: Standard 3**   \| \| --- \| --- \| --- \| |
| --- | --- | --- | --- |

**Supplemental Figure 3. TB-LAMP as a replacement test for smear microscopy in HIV-positives: Summary Receiver Operating Characteristic (SROC) curves.**

| \| **S3A: Standard 2**  **** \| **S3B: Standard 3**  **** \| \| --- \| --- \| \|  \| \| |
| --- | --- | --- | --- | --- |

**Supplemental Figure 4. TB-LAMP as an add-on test following smear microscopy: Summary Receiver Operating Characteristic (SROC) curves.**

| \| **S4A: Standard 1**   \| **S4B: Standard 2**   \| **S4C: Standard 3**   \| \| --- \| --- \| --- \| |
| --- | --- | --- | --- |

**Supplemental Figure 5. TB-LAMP vs. Xpert MTB/RIF: Forest plots of Xpert MTB/RIF diagnostic accuracy.**

**S5A. Standard 1**^1^


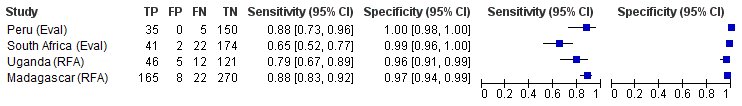


**S5B. Standard 2**^1^


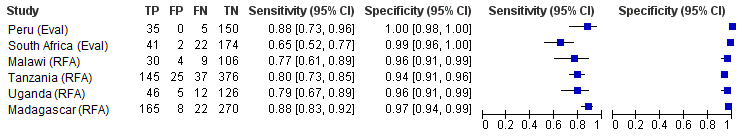


**S5C. Standard 3**^1^


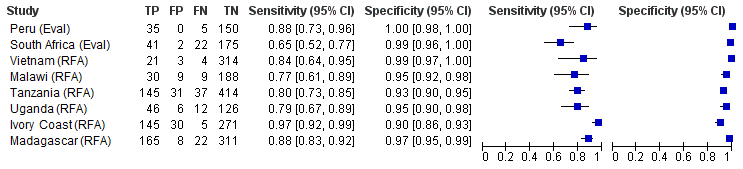


^1^Data restricted to study participants who had valid results for both TB-LAMP and Xpert MTB/RIF.

**Supplemental Figure 6. TB-LAMP vs. Xpert MTB/RIF: Forest plots of TB-LAMP diagnostic accuracy.**

**S6A. Standard 1**^1^


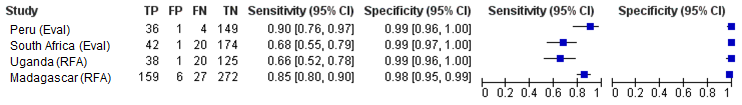


**S6B. Standard 2**^1^


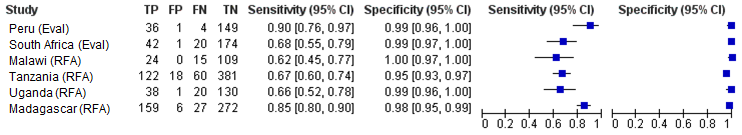


**S6C. Standard 3**^1^


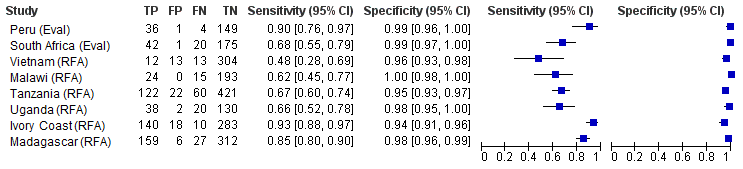


^1^Data restricted to study participants who had valid results for both TB-LAMP and Xpert MTB/RIF.

**Supplementary Figure 7. TB-LAMP vs. Xpert MTB/Rif: Forest plots of sensitivity difference.**

| **S7A: Standard 1^1^**  **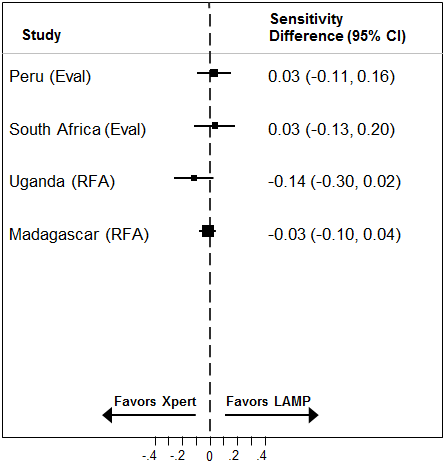** | **S7B: Standard 2^1^**  **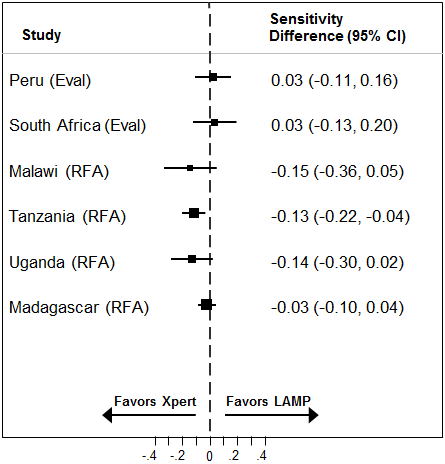** | **S7C: Standard 3^1^**  **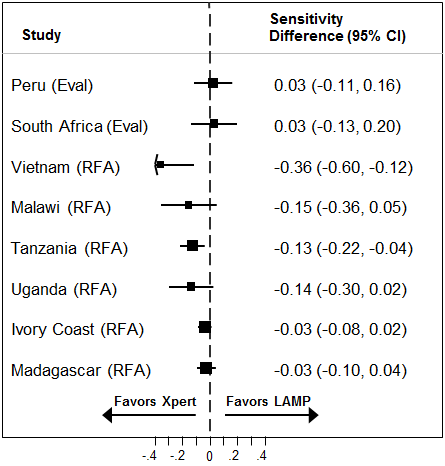** |
| --- | --- | --- |

^1^ Sensitivity difference x 100 = % sensitivity difference

**Supplementary Figure 8. TB-LAMP vs. Xpert MTB/Rif®: Forest plots of specificity difference.**

| **S8A: Standard 1^1^**  **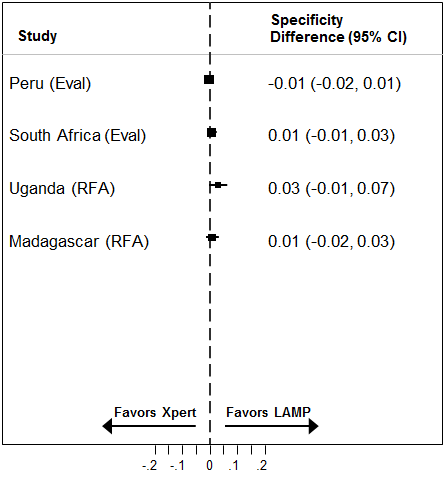** | **S8B: Standard 2^1^**  **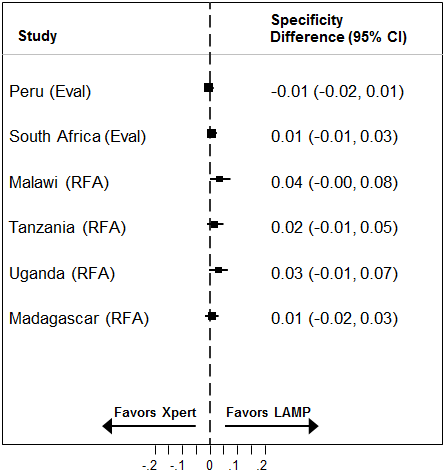** | **S8C: Standard 3^1^**  **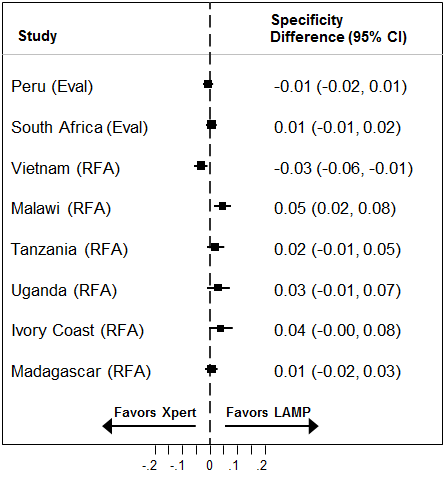** |
| --- | --- | --- |

^1^ Specificity difference x 100 = % sensitivity difference

**Supplementary Figure 9. Proportion of indeterminate TB-LAMP results.**

**
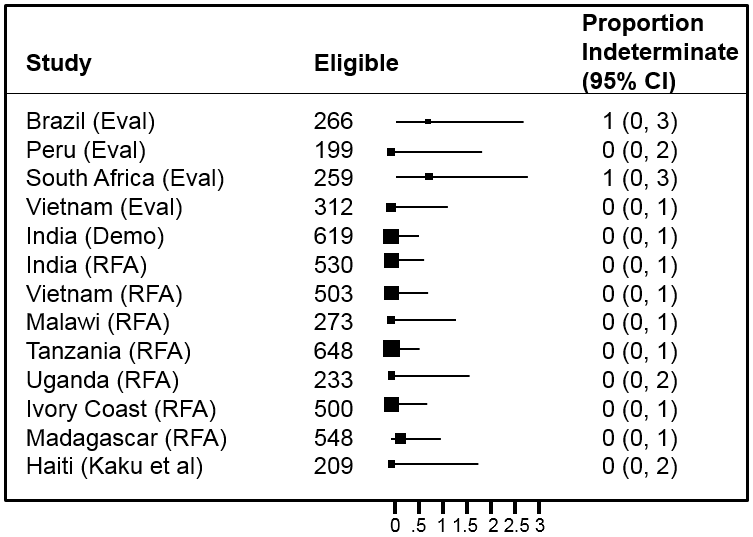
**

**Supplementary Table 1. Patients included for analysis.**

| **Study** | **Total Enrolled** | **TB-LAMP as a replacement test for smear microscopy in all eligible adults** | | | | **TB-LAMP as a replacement test for smear microscopy in all eligible HIV positive adults** | | | | **TB-LAMP as an add-on test following smear microscopy in smear-negative adults** | | | | **Comparison of diagnostic accuracy between TB-LAMP and Xpert** | | | |
| --- | --- | --- | --- | --- | --- | --- | --- | --- | --- | --- | --- | --- | --- | --- | --- | --- | --- |
|  |  | **Eligible^2^** | **Standard 1^1^** | **Standard 2^1^** | **Standard 3^1^** | **Eligible^3^** | **Standard 1^1^** | **Standard 2^1^** | **Standard 3^1^** | **Eligible^4^** | **Standard 1^1^** | **Standard 2^1^** | **Standard 3^1^** | **Eligible^5^** | **Standard 1^1^** | **Standard 2^1^** | **Standard 3^1^** |
| Brazil (EVAL) | 266 | 239 | 237  (99%) | 237  (99%) | 237  (99%) | 0 | --- | --- | --- | 182 | 180  (99%) | 180  (99%) | 180  (99%) | --- | --- | --- | --- |
| Peru (EVAL) | 199 | 198 | 198  (100%) | 198  (100%) | 198  (100%) | 0 | --- | --- | --- | 173 | 173  (100%) | 173 (100%) | 173  (100%) | 190 | 190  (100%) | 190  (100%) | 190  (100%) |
| South Africa (EVAL) | 259 | 240 | 237  (99%) | 237  (99%) | 238  (99%) | 83 | 82  (99%) | 82  (99%) | 83  (100%) | 207 | 204  (99%) | 204  (99%) | 205  (99%) | 238 | 237  (99%) | 237  (99%) | 238  (100%) |
| Vietnam (EVAL) | 312 | 304 | 304  (100%) | 304  (100%) | 304  (100%) | 0 | --- | --- | --- | 186 | 186  (100%) | 186  (100%) | 186  (100%) | --- | --- | --- | --- |
| India (DEMO) | 619 | 598 | --- | 559  (94%) | 586  (98%) | 0 | --- | --- | --- | 432 | --- | 403  (93%) | 421  (98%) | --- | --- | --- | --- |
| India (RFA) | 530 | 504 | --- | --- | 446  (89%) | 0 | --- | --- | --- | 0 | --- | --- | --- | --- | --- | --- | --- |
| Vietnam (RFA) | 503 | 364 | --- | --- | 361  (99%) | 0 | --- | --- | --- | 351 | --- | --- | 348  (99%) | 344 | --- | --- | 342  (99%) |
| Malawi (RFA) | 273 | 265 | --- | 149  (56%) | 234  (88%) | 113 | --- | 62  (55%) | 100  (89%) | 0 | --- | --- | --- | 258 | --- | 148  (57%) | 232  (90%) |
| Tanzania (RFA) | 648 | 648 | --- | 587  (91%) | 632  (98%) | 38 | --- | 36  (95%) | 38  (100%) | 489 | --- | 438  (90%) | 478  (98%) | 630 | --- | 581  (92%) | 625  (99%) |
| Uganda (RFA) | 233 | 190 | 184  (97%) | 189  (99%) | 190  (100%) | 92 | 87  (95%) | 91  (99%) | 92  (100%) | 149 | 149  (100%) | 149  (100%) | 149  (100%) | 190 | 184  (97%) | 189  (99%) | 190  (100%) |
| Ivory Coast (RFA) | 500 | 480 | --- | --- | 451  (94%) | 59 | --- | --- | 57  (97%) | 329 | --- | --- | 305  (93%) | 480 | --- | --- | 451  (94%) |
| Madagascar (RFA) | 548 | 521 | 476  (91%) | 476  (91%) | 516  (99%) | 0 | --- | --- | --- | 350 | 333  (95%) | 333  (95%) | 347  (99%) | 507 | 464  (92%) | 464  (92%) | 504  (99%) |
| Haiti (Kaku et al) | 209 | 209 | 174  (83%) | 174  (83%) | 203  (97%) | 0 | --- | --- | --- | 124 | 124  (100%) | 124  (100%) | 124  (100%) | --- | --- | --- | --- |
| **TOTAL** | **5099** | **4760** | **1810**  **(38%)** | **3110**  **(65%)** | **4596**  **(97%)** | **385** | **169**  **(44%)** | **271**  **(70%)** | **370**  **(96%)** | **2972** | **1349**  **(45%)** | **2190**  **(74%)** | **2916**  **(98%)** | **2837** | **1075**  **(38%)** | **1809**  **(64%)** | **2772**  **(98%)** |

--- indicates that reference standard criteria were not met by at least 5 TB and 5 non-TB patients

^1^All reference standards classify patients as having TB if ≥1positive culture was confirmed as M. tuberculosis by speciation testing. To be classified as not having TB, patients were required to have no positive and at least 1) two negative cultures on two different sputum specimens (Standard 1); 2) two negative cultures on the same or different sputum specimens (Standard 2); or 3) at least one negative culture (Standard 3).

^2^Total eligible includes missing and indeterminate LAMP results: Brazil EVAL (n=2 indeterminate), South Africa EVAL (n=2 indeterminate), Malawi RFA (n=5 missing), Tanzania RFA (n=11 missing), Madagascar RFA (n=1 missing, n=1 indeterminate), Haiti (n=6 missing).

^3^Total eligible includes missing and indeterminate LAMP results: South Africa EVAL (n=1 indeterminate), Malawi RFA (n=3 missing).

^4^Total eligible includes missing and indeterminate LAMP results: Brazil EVAL (n=2 indeterminate), South Africa EVAL (n=2 indeterminate), Tanzania RFA (n=6 missing), Madagascar RFA (n=1 indeterminate).

^5^Total eligible includes only patients with positive/negative TB-LAMP and Xpert MTB/RIF results

**Supplementary Table 2. Signaling questions for QUADAS-2 domains.**

| **Study** | **Patient Selection^1^** | | | | **Index Test (TB-LAMP)^1^** | | | **Reference Standard^1^** | | | | **Flow and Timing^1^** | | |
| --- | --- | --- | --- | --- | --- | --- | --- | --- | --- | --- | --- | --- | --- | --- |
|  | **Risk of Bias** | | **Applicability Concerns** | | | **Risk of Bias** | | **Applicability Concerns** | | **Risk of Bias** | | | **Risk of Bias** | |
|  | **Were individuals with a prior history of TB excluded?** | **Was a consecutive sample of participants enrolled?** | **Were inclusion and exclusion criteria known?** | **Did the study enroll a representative population?** | | **Were TB-LAMP operators blinded to smear and Xpert results?** | **Were indeterminate/invalid results reported?** | **Was the standard TB-LAMP protocol followed?** | **Were at least 2 separate samples used for cultures?** | **Was at least 1 MGIT done?** | **Were there acceptable culture contamination rates (5-10%) for MGIT?** | | **Was a study participant flow diagram available?** | **Were less than 20% of participants excluded from best possible reference standard?^8^** |
| Brazil EVAL | Yes | Yes | Yes | No^3^ | | Yes | Yes | Yes | Yes | Yes | No^6^ | | Yes | Yes (S1) |
| Peru EVAL | Yes | Yes | Yes | No^3^ | | Yes | Yes | Yes | Yes | Yes | No^6^ | | Yes | Yes (S1) |
| South Africa EVAL | Yes | Yes | Yes | No^3^ | | Yes | Yes | Yes | Yes | Yes | No^6^ | | Yes | Yes (S1) |
| Vietnam EVAL | Yes | Yes | Yes | No^3^ | | Yes | Yes | Yes | Yes | Yes | No^6^ | | Yes | Yes (S1) |
| India DEMO | Yes | Yes | Yes | Yes | | Yes | Yes | Yes | No^5^ | Yes | Yes | | Yes | Yes (S2) |
| India RFA | Unclear^2^ | Yes | Yes | Yes | | Yes | Yes | Yes | No^5^ | Yes | No^7^ | | Unclear | Yes (S3) |
| Vietnam RFA | Unclear^2^ | Yes | Yes | Yes | | Yes | Yes | Yes | No^5^ | Yes | No^6^ | | Unclear | Yes (S3) |
| Malawi RFA | Yes | Yes | Yes | Yes | | Yes | Yes | Yes | No^5^ | Yes | Yes | | Yes | No (S2) |
| Tanzania RFA | Unclear^2^ | Yes | Yes | No^4^ | | Yes | Yes | Yes | No^5^ | Yes | Yes | | Unclear | Yes (S2) |
| Uganda RFA | Unclear^2^ | Yes | Yes | Yes | | Yes | Yes | Yes | Yes | Yes | No^6^ | | Unclear | Yes (S1) |
| Ivory Coast RFA | Yes | Yes | Yes | Yes | | Yes | Yes | Yes | No^5^ | Yes | Yes | | Unclear | Yes (S3) |
| Madagascar RFA | Yes | Yes | Yes | Yes | | Yes | Yes | Yes | Yes | No | No^6^ | | Unclear | Yes (S1) |
| Haiti (Kaku el al) | Unclear^2^ | Yes | Yes | Yes | | Yes | Yes | Yes | Yes | Yes | No^6^ | | Unclear | Yes (S1) |

Abbreviations: TB- tuberculosis; MGIT – Mycobacterial Growth Indicator Tube; S1 – Standard 1; S2 – Standard 2; S3 – Standard 3

### ^1^The QUADAS-2 tool was used to assess methodological quality of each study included (3). Specific yes/no signaling questions were tailored for each QUADAS-2 domain (patient selection, index test [TB-LAMP], reference standard, and study flow and timing). For each domain, the risk of bias or applicability concern for an individual study was ranked as low if the answer to all signaling questions was “Yes”, high if the answer to one or more signaling questions was “No”, or unclear if the answer to one or more signaling questions was “Unclear”

^2^Study excluded patients on TB treatment within 60 days of enrollment but patients with any history of prior TB were not excluded and information on prior TB history was not reported to enable exclusion of such patients from the analysis. Molecular assays such as TB-LAMP can detect persisting DNA from non-viable bacteria, leading to false-positive results.

^3^Study was conducted at a reference laboratory, which typically has a different population and more experienced staff than lower-level health centers where TB-LAMP would be used as a replacement test for sputum smear microscopy.

^4^Patient eligibility was determined by a pulmonary specialist. Clinicians referred patients they thought could have TB to a pulmonary specialist. The pulmonary specialist ultimately decided whether or not the patient should be presumed to have TB and therefore enrolled into the study.

^5^Study performed 2 cultures from 1 sample (India DEMO, Malawi RFA, Tanzania RFA) or 1 culture from 1 sample (India RFA, Ivory Coast RFA, Vietnam RFA)

^6^MGIT contamination rate was <5%. Excessive decontamination can impede recovery of MTB from culture(22,23).

^7^MGIT contamination rate was >10%. Insufficient decontamination can impeded recovery of MTB from culture(22,23).

^8^Based on analysis of TB-LAMP accuracy among all adults

**Supplementary Table 3. TB-LAMP as a replacement test for smear microscopy: Exploration of heterogeneity.**

| **Study sub-group** | **Sensitivity** | | **Specificity** | |
| --- | --- | --- | --- | --- |
|  | **Pooled estimate** | **Heterogeneity** | **Pooled estimate** | **Heterogeneity** |
| Reference labs^1^ | 78.0 (68.9—85.0) | I^2^ 74%, p=0.009 | 97.8 (92.2—99.4) | I^2^ 80%, p=0.002 |
| Hospital-affiliated clinics^2^ | 86.8 (67.9—95.4) | I^2^ 96%, p<0.001 | 97.5 (95.3—98.6) | I^2^ 76%, p=0.001 |
| Microscopy centers^3^ | <4 studies | <4 studies | <4 studies | <4 studies |
| High quality studies for patient selection^4^ | 84.2 (71.1—92.0) | I^2^ 83%, p<0.001 | 98.1 (94.3—99.4) | I^2^ 87%, p<0.001 |
| High quality studies for reference standard | <4 studies | <4 studies | <4 studies | <4 studies |

^1^ Brazil EVAL, Peru EVAL, South Africa EVAL, Vietnam EVAL

^2^ India RFA, Tanzania RFA, Uganda RFA, Ivory Coast RFA, Madagascar RFA, Haiti Kaku et al

^3^ India DEMO, Vietnam RFA, Malawi RFA

^4^ India DEMO, Ivory Coast RFA, Madagascar RFA, Malawi RFA

**Supplementary Figure Legends**

**Supplementary Figure 1. Forrest plots of TB-LAMP diagnostic accuracy, additional reference standards.** The figures show the sensitivity and specificity of TB-LAMP in individual studies in reference to all additional reference standards not judged best available for TB-LAMP as a replacement test for smear microscopy in all patients (Panel S1A and Panel S1B), TB-LAMP as a replacement test for smear microscopy in HIV-positive adults (Panel S1C), and TB-LAMP as an add-on test following smear microscopy (Panel S1D and Panel S1E). All reference standards classify patients as having TB if ≥1 positive culture was confirmed as *M. tuberculosis* by speciation testing. To be classified as not having TB, patients were required to have no positive and at least 1) two negative cultures on two different sputum specimens (Standard 1); 2) two negative cultures on the same or different sputum specimens (Standard 2); or 3) at least one negative culture (Standard 3). Visual inspection of all three forest plots indicates considerable heterogeneity in sensitivity estimates but less heterogeneity in specificity estimates.

**Supplementary Figure 2. TB-LAMP as a replacement for sputum smear microscopy: Summary Receiver Operating Characteristic (SROC) curves.** The figure shows SROC curves for TB-LAMP (green line), individual study estimates (grey circle), pooled estimates (red square), and the 95% confidence region for pooled estimates (yellow dotted line) when using 3 culture-based reference standards. All reference standards classify patients as having TB if ≥1positive culture was confirmed as *M. tuberculosis* by speciation testing. To be classified as not having TB, patients were required to have no positive and at least 1) two negative cultures on two different sputum specimens (Standard 1); 2) two negative cultures on the same or different sputum specimens (Standard 2); or 3) at least one negative culture (Standard 3).

**Supplemental Figure 3. TB-LAMP as a replacement test for smear microscopy in HIV-positives: Summary Receiver Operating Characteristic (SROC) curves.** The figure shows SROC curves for TB-LAMP (green line), individual study estimates (grey circle), pooled estimates (red square), and the 95% confidence region for pooled estimates (yellow dotted line) when using 2 culture-based reference standards (no studies qualified for Standard 1). All reference standards classify patients as having TB if ≥1positive culture was confirmed as *M. tuberculosis* by speciation testing. To be classified as not having TB, patients were required to have no positive and at least 1) two negative cultures on the same or different sputum specimens (Standard 2); or 2) at least one negative culture (Standard 3).

**Supplemental Figure 4. TB-LAMP as an add-on test following smear microscopy: Summary Receiver Operating Characteristic (SROC) curves.** The figure shows SROC curves for TB-LAMP (green line), individual study estimates (grey circle), pooled estimates (red square), and the 95% confidence region for pooled estimates (yellow dotted line) when using 3 culture-based reference standards. All reference standards classify patients as having TB if ≥1positive culture was confirmed as *M. tuberculosis* by speciation testing. To be classified as not having TB, patients were required to have no positive and at least 1) two negative cultures on two different sputum specimens (Standard 1); 2) two negative cultures on the same or different sputum specimens (Standard 2); or 3) at least one negative culture (Standard 3).

**Supplemental Figure 5. TB-LAMP vs. Xpert MTB/RIF: Forest plots of Xpert MTB/RIF diagnostic accuracy.** The figure shows forest plots of Xpert MTB/RIF sensitivity and specificity in reference to 3 culture-based reference standards for individual studies. All reference standards classify patients as having TB if ≥1positive culture was confirmed as *M. tuberculosis* by speciation testing. To be classified as not having TB, patients were required to have no positive and at least 1) two negative cultures on two different sputum specimens (Standard 1); 2) two negative cultures on the same or different sputum specimens (Standard 2); or 3) at least one negative culture (Standard 3).

**Supplemental Figure 6. TB-LAMP vs. Xpert MTB/RIF: Forest plots of TB-LAMP diagnostic accuracy.** The figure shows forest plots of TB-LAMP sensitivity and specificity in reference to 3 culture-based reference standards for individual studies. All reference standards classify patients as having TB if ≥1positive culture was confirmed as *M. tuberculosis* by speciation testing. To be classified as not having TB, patients were required to have no positive and at least 1) two negative cultures on two different sputum specimens (Standard 1); 2) two negative cultures on the same or different sputum specimens (Standard 2); or 3) at least one negative culture (Standard 3).

**Supplementary Figure 7. TB-LAMP vs. Xpert MTB/Rif: Forest plots of sensitivity difference.** The figure shows forest plots of the sensitivity difference between TB-LAMP and Xpert MTB/Rif® for individual studies. The sensitivity of both tests was calculated in reference to 3 culture-based reference standards. All reference standards classify patients as having TB if ≥1positive culture was confirmed as *M. tuberculosis* by speciation testing. To be classified as not having TB, patients were required to have no positive and at least 1) two negative cultures on two different sputum specimens (Standard 1); 2) two negative cultures on the same or different sputum specimens; or 3) at least one negative culture (Standard 3). Visual inspection of forest plots and statistical testing indicate minimal heterogeneity with Standard 1 (*I*^2^ 0%, p=0.41), some heterogeneity with Standard 2 (*I*^2^ 34%, p=0.18), and significant heterogeneity with Standard 3 (*I*^2^ 55%, p=0.03).

**Supplementary Figure 8. TB-LAMP vs. Xpert MTB/Rif®: Forest plots of specificity difference.** The figure shows forest plots of the specificity difference between TB-LAMP and Xpert MTB/Rif® for individual studies. The specificity of both tests was calculated in reference to 3 culture-based reference standards. All reference standards classify patients as having TB if ≥1positive culture was confirmed as *M. tuberculosis* by speciation testing. To be classified as not having TB, patients were required to have no positive and at least 1) two negative cultures on two different sputum specimens (Standard 1); 2) two negative cultures on the same or different sputum specimens (Standard 2); or 3) at least one negative culture (Standard 3). Visual inspection of forest plots and statistical testing indicate minimal heterogeneity with Standard 1 (*I*^2^ 28%, p=0.25) and Standard 2 (*I*^2^ 37%, p=0.16), but significant heterogeneity with Standard 3 (*I*^2^ 72%, p=0.001).

**Supplementary Figure 9. Proportion of indeterminate TB-LAMP results.** The figure shows a forest plot of the proportion of indeterminate TB-LAMP results among all adults for individual studies. Visual inspection of forest plots and statistical testing indicate minimal heterogeneity (*I*^2^ 28%, p=0.25).
